# Integrating Wearable Technology in Digital Therapy for People With Psychosis (SloMo): Mixed Methods Co-Design and Feasibility Study

**DOI:** 10.2196/91188

**Published:** 2026-10-02

**Authors:** Kathryn M Taylor, Thomas Ward, Matteo Cella, Philippa Garety, Amy Grant, Katie Philps, Anna Wojdecka, Melanie Flory, Amy Hardy

**Affiliations:** 1 Department of Psychology, Institute of Psychiatry, Psychology & Neuroscience, King's College London, Henry Wellcome Building, IoPPN, 16 De Crespigny Park, London, SE5 8AF, United Kingdom, +44 20 7848 0002; 2 South London and Maudsley NHS Foundation Trust, London, United Kingdom; 3 Research and Development, Sussex Partnership NHS Foundation Trust, Sussex, United Kingdom; 4 Helen Hamlyn Centre for Design, Royal College of Art, London, United Kingdom

**Keywords:** human-centered design, mHealth, paranoia, schizophrenia, wearable electronic devices, psychotic disorders, digital health, feasibility studies, user-centered design, mobile health

## Abstract

**Background:**

Digital health interventions for psychosis, like SloMo, leverage smartphone technology to help transfer learning from therapy to real-life situations. Usage relies on motivation, recall, and awareness. Wearable devices that track physiological signs of stress can boost engagement by encouraging the use of coping strategies when most needed.

**Objective:**

This study aims to co-design an integrated user interface for wearable technology and the SloMo mobile app, and explore its usability, acceptability, engagement, and preliminary clinical outcomes for individuals with psychosis through a feasibility study.

**Methods:**

A co-design team developed the wearable augmented SloMo app using the Double Diamond framework. The team included 10 experts by experience based across three UK National Health Service (NHS) mental health trusts, who had completed SloMo as part of a randomized controlled trial, and were purposively recruited to ensure a range of demographic backgrounds. Users tested the app for 8 consecutive weeks during a 5-month data collection period in 2018, providing feedback that guided iterative improvements. Self-reported engagement, usability, and acceptability were collected through quantitative ratings and interviews, evaluated through qualitative content analysis. Assessments of paranoia and well-being were completed pre- and post use, summarized descriptively, with change scores calculated and evaluated for reliable change.

**Results:**

The Spire Stone clothing-adhered biosensor, which monitors respiratory rate and stress physiology, was chosen to augment SloMo. A minimum viable product of an integrated interface was developed. Feedback identified that users needed the technology to be easy to use, secure, and tailored to them through user control. Eight (8/10, 80%) users reported that the wearable prompted increased use of the SloMo app’s features and other coping strategies. Self-reported engagement varied according to EBE user experience of the technology: higher use of the technology (n=3) was associated with higher usefulness (25% reduction in paranoia, SD 6%) and acceptability (84% satisfaction, SD 13%). Conversely, individuals that engaged with the device less (n=7) did not find it as useful (11% increase in paranoia, SD 17%) and were only modestly satisfied (63%, SD 20%).

**Conclusions:**

Wearable technology is acceptable and can be integrated with a digitally supported therapy for psychosis to promote engagement and potentially support management of difficulties. However, further development work is needed to ensure acceptability and usefulness across a broader range of user needs.

## Introduction

SloMo is a digitally supported cognitive behavioral therapy for psychosis (CBTp) that reduces paranoia [1]. It was developed to address the limitations of traditional psychological therapies for psychosis in relation to access, experience, and outcomes [2]. SloMo achieves this through targeting fast-thinking habits related to jumping to conclusions and belief inflexibility biases, which are established causal mechanisms in paranoia [3,4]. User experience is improved through co-design to leverage the capabilities of digital technologies [2,5]. SloMo consists of one-to-one sessions with a trained therapist, supported by a web-based therapy platform. Between sessions, the mobile app supports the individual to implement strategies independently, therefore providing continuity between therapy and daily life.

Between session practice, or “homework,” is a fundamental component of CBTp [6], facilitating the generalization of therapeutic learning into real-life contexts. Such practice has shown to be predictive of successful outcomes in cognitive behavioral therapy (CBT) [7]. The availability, portability, and acceptability of smartphones means mobile apps are well placed to support skill practice in everyday life [8]. However, completion of tasks between sessions requires motivation, recall, understanding, and perceived benefits [9]. Difficulties in self-initiated behavior and planning can prevent effective use of strategies [10]. Relatedly, early experimental evaluations of SloMo showed its effects were moderated by cognitive difficulties, such as working memory problems, and negative symptoms [4].

Elevated levels of stress and anxiety present a further barrier to task completion, by detrimentally impacting functioning and cognition [11]. Early feasibility testing of SloMo demonstrated that individuals had greater difficulty remembering to use SloMo between sessions when distressed, and desired discrete prompts to help monitor their stress and encourage use of strategies [2]. User engagement or adherence, the extent to which users are willing to use and continue to use an intervention as intended, may influence effectiveness [12]. Consequently, approaches to improving engagement have been a significant focus to realizing the potential of digital health interventions and progress real-world implementation [13-15].

In psychosis, physiological parameters, such as heart rate variability, can provide an indicator of the onset of worsening paranoia [16]. Detection of elevated stress, anxiety, and paranoia could therefore prompt use of relaxation techniques or other coping strategies available via SloMo. However, consistent self-monitoring of stress can be challenging and effortful. Difficulties in identification of emotional states (ie, alexithymia) are common for people with psychosis [17]. Wearable devices provide a potential solution for detecting and managing stress through continuous passive monitoring of physiological signals. Accompanied by biofeedback and guidance via the technology or a linked device (eg, smartphone), this can support behavior change before stress escalates [18,19]. For SloMo users, notification of elevated physiological signs of stress could increase emotional awareness, providing opportunity to interrupt fast thinking habits, and support use of alternative slower thinking or coping strategies.

To be used and successfully integrated into daily life, wearable technologies must be usable, useful, and acceptable to the user [20]. Usability refers to the ease of use of the technology, including both the device itself and the app interface. Usefulness is the extent to which the device can achieve its intended purpose, ie, accurate monitoring and supporting self-management of difficulties. Acceptability encompasses factors that impact the user’s willingness to use the technology, such as comfort, perceived value, and enjoyment. Poor usability risks impeding rather than augmenting outcomes [14]. This is of particular importance for individuals from marginalized groups who may be more likely to experience technology-related inequalities in access, use, and confidence [21]. For people with severe mental health needs, for whom privacy and data misuse concerns are common, security and trustworthiness of wearable technologies are especially important [22].

Inclusive, human-centered co-design (iHCD) is a method that places the user at the center of the design process: the needs of users representing a wide range of characteristics, abilities, and perspectives are explored, and design solutions incorporating these are iteratively tested and improved [23,24]. Transdisciplinary iHCD that includes experts by experience (EBE, ie, individuals, such as service users or carers, who have lived experience of the target problem), enhances adherence to digital interventions [25], promotes ethical usage [26], and is crucial for ensuring technologies are fit for purpose and meet the needs of diverse users [5]. This study adopted the Double Diamond process, a Human-Centered Design (HCD) framework using inclusive people-centered approaches [27,28].

The aims of this study were to (1) describe the development of a minimum viable product for an integrated user interface designed for wearable technology and the SloMo mobile app through co-design; and (2) conduct a feasibility study to explore usability, acceptability, engagement, and preliminary clinical outcomes of the developed technology, for individuals with psychosis.

## Methods

This study was nested within the SlowMo randomized controlled trial (RCT) [1]. The first version of the therapy was “SlowMo,” however spelling has since been updated to “SloMo” in line with feedback from individuals with lived experience of psychosis [5]. The RCT is referred to as SlowMo and the therapy as SloMo here forward.

### Co-Design Team

A project team consisting of experts by experience (n=10), designers (n=4), software developers (n=5), clinicians (n=6), and researchers (n=5) participated in the co-design.

Ten individuals, who had previously received SloMo therapy as part of the trial and therefore had experience of the SloMo software, were invited to participate as EBE Co-Designers. In accordance with iHCD, purposive, and stratified sampling was applied with the aim of ensuring representation across trial sites, ethnicity, age, and levels of technology confidence. EBEs were recruited from the 3 trial sites of London, Sussex, and Oxford. Eligible participants for the trial were: 18 years or older; had persistent (≥3 months) distressing paranoia; a diagnosis of schizophrenia spectrum psychosis; capacity to provide informed consent; and sufficient English to participate in trial processes. Participants were excluded if they had profound visual or hearing impairment, were unable to engage in assessments, were currently receiving psychological therapy for paranoia, or had a primary diagnosis of substance use disorder, personality disorder, organic syndrome, or learning disability. For further details of eligibility assessment, refer to Garety et al (2021) [1]. EBEs were invited to participate in this study following completion of all RCT research procedures (ie, 24-week follow-up assessment).

Characteristics of the EBEs are reported in Table 1. At baseline, persecutory ideation severity ranged from average (22) to very severe (62; see Measures: Green Paranoid Thought Scale [GPTS] [29]). Well-being scores were typically lower than the general population average (range 31 to 51; Warwick-Edinburgh Mental Wellbeing Scale [WEMWBS] [30]). Participant data from the RCT was extracted to facilitate description of the group and to inform preliminary understanding of who may benefit from SloMo augmentation with wearable technology. All EBEs completed all 8 sessions of SloMo therapy. Confidence and frequency of smartphone use (excluding calls) before SloMo were rated from 0 (Not at all; Never) to 100 (Totally; All the time). Confidence ranged from 11 to 95 (mean 67.0, SD 26.8), and frequency from 10 to 100 (mean 70.6, SD 27.6). In the RCT, a clinically observable response to SloMo therapy (defined as a 20% improvement in symptoms [31]) was seen for six (6/10; 60%) for paranoia (total GPTS), and two (2/10; 20%) for well-being (WEMWBS). The EBEs therefore represented a diverse subgroup of those who had completed SloMo therapy, with regards to demographics and technology attitudes and behaviors.

**Table 1.** Baseline demographic and clinical characteristics of EBE^a^ co-designers (n=10).

Sociodemographic characteristic	Values
Age in years, mean (SD)	43.8 (11.0)
Sex, n (%)	
Male	8 (80.0)
Female	2 (20.0)
Ethnicity, n (%)	
Black, Black British, Black Welsh, Caribbean, or African	4 (40.0)
White	4 (40.0)
Asian, Asian British, Asian Welsh	1 (10.0)
Other ethnic group	1 (10.0)
GPTS^b^, mean (SD)	
Total	76.1 (27.0)
Part A - referential ideas	37.8 (13.4)
Part B - persecution	38.3 (14.1)
WEMWBS^c^, mean (SD)	
Total	39.7 (11.2)

a EBE: expert by experience co-designer.

b GPTS: Green et al [29] Paranoid Thought Scale.

c WEMWBS: Warwick-Edinburgh Mental Wellbeing Scale.

### Intervention: SloMo

In SloMo therapy, an online therapy platform, accessed using a laptop or tablet, is used during one-to-one sessions with a trained therapist. A native Android mobile app supports strategy practice between sessions, with reminders and individualized content. An Android device, with the SloMo app installed, was provided to all participants for participation in the RCT and this study. This study included use of the mobile app software only, as EBEs had completed the full SloMo therapy prior to their involvement. Due to the early stage of development, access to the SloMo app was restricted to the supplied Android handset only, and was not available on the participant’s own smartphone or alternative devices (eg, iOS).

The SloMo mobile app is described in detail elsewhere [2]. Key features include (1) a home screen where users can view personalized worries and safer thoughts; (2) a “Slow Down” function in which users are encouraged to notice worries, and use SloMo tips to help them to slow down to find ways of feeling safer; (3) a summary of therapy sessions and learning through “My Journey”; and (4) two behavioral coping enhancement activities: FlowMo, in which users are visually guided through an exercise to promote slow-paced breathing, with an expanding and shrinking bubble, and GoMo, a distraction activity in which users are tasked with popping bubbles by tapping as they appear. FlowMo and GoMo were novel additions to the SloMo app, providing opportunity to intervene in the moment in response to elevated stress.

### Ethical Considerations

The study was granted ethical approval through notice of substantial amendment of the RCT, which was reviewed and approved by the National Health Service Health Research Authority and London-Camberwell St. Giles Research Ethics Committee (16/LO/1862). After completion of all RCT procedures, eligible participants were provided with an information sheet and gave written, informed consent for participation in this substudy. Deidentified participant research data were held separately to personally identifiable information on secure servers accessible only to those in the research team. Participants were reimbursed £20 (GBP £1=US $1.43 as of February 1, 2018) for each meeting attended.

### Procedure

Enrolment to the substudy commenced an average of 52 (SD 32) days after the SlowMo RCT 24-week follow-up assessment meeting, and 135 (SD 36) days after the posttherapy assessment.

#### Co-Design Procedures

The 4-phase Double Diamond framework [27], outlined below, was followed using a hybrid waterfall-agile approach, appropriate to the context of digital healthcare [5]. Following selection of the device, EBEs tested the technology for an 8-week period, contributing to all stages of the Double Diamond. The data collection period lasted 5 months, between February and July 2018.

##### Discover

The “Discover” phase is a divergent process focused on developing a broad understanding of the problem from multiple perspectives, while being informed by the existing evidence base. Initially, this phase consisted of “desk research” led by the designers, reviewing the existing market of wearable technologies to select an appropriate device for the augmentation of SloMo. Appropriateness was considered according to existing empirical evidence of acceptability to users, stress monitoring performance, feasibility of integration with SloMo, and compatibility with the previously identified user requirements for SloMo [2]. These prior co-design insights from SloMo therapy also informed the development of an initial wearable integration interface prototype. Designers, developers, and clinicians conducted testing of the wearable technology and prototype performance to identify improvements and bug fixes necessary before EBE testing.

EBEs were provided with the chosen wearable technology, shown how to use it, and asked to wear the device during the day over an 8-week period. Each EBE was provided a smartphone handset set-up with their own user profile from SloMo therapy (ie, tailored according to their own previously identified worries and safer thoughts). They were informed that they would receive feedback regarding stress levels through the mobile device. Handsets had a wireless internet connection for data transfer during the period of their involvement. Initial insights into attitudes and experience of the wearable were obtained through qualitative feedback at baseline and after one week of use. The EBEs also completed measures of paranoia, well-being, and were asked about attitudes towards monitoring stress and using technology.

EBEs completed the initial meeting with a study research worker at a time and location convenient to the individual, such as at home, their care team base, or at the research premises. Each meeting lasted up to one hour. If preferred, after initial set-up, interim qualitative feedback was collected remotely via telephone call. EBEs were asked at each meeting if they consented to audio recording, and written notes were produced by the research worker subsequently to summarize their feedback. If consent was not provided, the research worker took detailed written notes during the meeting. Questionnaire measures were completed on paper, with the support of the research worker as needed.

##### Define

The “Define” phase is a convergent process in which insights from the Discover phase were reviewed by the developers, designers, clinicians, and researchers, and synthesized to articulate user needs for the SloMo-wearable interface and a preliminary design brief for the solution. Throughout the co-design process, the study lead (AH) was responsible for the collation of EBE insights and ensuring user needs were refined accordingly, in collaboration with the interdisciplinary team.

##### Develop

The “Develop” phase is a divergent process in which a wide range of ideas for addressing the design brief are considered. Early-stage prototype solutions are iteratively developed, tested, and refined. Initial ideas in this phase were generated by the designers, software developers, and researchers. A series of software development sprints facilitated the iterative implementation of preferred solutions into the interface prototype, shaped by the ongoing collection of qualitative feedback from EBEs running in parallel. Decisions regarding which solutions should be kept were informed by both quantitative (ie, frequency of shared opinion) and qualitative content of feedback.

##### Deliver

In the “Deliver” phase, a convergent process is employed to develop the optimized solution into a higher fidelity prototype as the “minimum viable product” for further testing and release. The prototype was then tested and evaluated by the EBE group in a feasibility study. Usability and acceptability of the optimized interface were evaluated qualitatively and quantitatively through interviews and a user experience survey (UES). EBEs undertook reassessments of paranoia and well-being to support preliminary evaluation of the potential usefulness of the technology.

### Measures

#### Stress Monitoring and Technology Use Attitudes

A semistructured interview was conducted during the Discover phase to understand the EBEs’ attitudes to monitoring stress and using technology (see Section 1 in Multimedia Appendix 1).

#### Usability and Acceptability

##### Semistructured Interview

Acceptability of the wearable device integration for SloMo was assessed at each follow-up meeting. Qualitative data regarding EBEs’ views (ie, perceived helpfulness, concerns, and suggestions for improvement) and usage (ie, self-reported engagement; in what ways it is or is not being used, and likelihood of continued use) of the Spire Stone (Spire Health) and the SloMo app were elicited through a semistructured interview (Section 2 in Multimedia Appendix 1).

##### User Experience

User experience was assessed by the UES (see Multimedia Appendix 1; adapted from Ben-Zeev et al [32], as used in the SloMo RCT [1]). The UES is a 10-item measure consisting of 3 subscales assessing usefulness, usability, and enjoyment. Items are rated on a 0 (totally disagree) to 10 (totally agree) scale. Ratings are summed for each subscale and a percentage score calculated. The total score represents an overall measure of user satisfaction.

### Usefulness

#### Paranoia

Paranoia was assessed using the GPTS [29]. The GPTS consists of two 16-item self-report subscales: part A—assessing referential ideas; part B—persecutory ideation. Items are rated on a 5-point scale (range 16 to 80 for each scale), with higher scores indicating greater difficulty. Internal reliability of the GPTS is considered excellent, with a reported Cronbach α of >0.9 [29,33].

#### Well-Being

Well-being was assessed using the WEMWBS [30], a 14-item self-report scale. Items are rated on a 5-point scale (range 14 to 70), with higher scores indicating better well-being. Very strong internal reliability has been reported (Cronbach α=0.93; [33]). A score of 42.9 or lower has been used to indicate poor psychological well-being [33].

### Data Analysis

A combination of conventional and directed approaches to qualitative content analysis [34] was used to summarize usability and acceptability feedback. The lead author (AH) first read and reread all feedback for familiarization with the data. Using NVivo software (Lumivero), key details related to the EBE’s user experience of the technology was highlighted. Preliminary codes were generated inductively and refined through iteration. Final codes were organized into categories according to whether they related to usability, acceptability, or usefulness. Frequency of participant responses for each code supported development decisions. Following identification of patterns during the initial inductive coding phase, qualitative feedback reflecting the extent of use of the technology was coded to indicate “lower,” “mid,” or “higher” engagement (see Table S1 in Multimedia Appendix 1).

Descriptive statistics were reported as frequency n (%) for categorical variables and mean (SD) for continuous variables. Change scores were calculated for paranoia and well-being outcomes between baseline and 8-week follow-up and evaluated for reliable change and a clinically observable response. The reliable change index (RCI) was calculated using the equation described by Jacobsen and Traux [35] (see Table S2 in Multimedia Appendix 1). A 20% reduction in symptoms was considered to represent a “clinically observable response,” as has been used previously to signify at least “minimal improvement” [31]. An explorative subgroup analysis was conducted to investigate patterns of engagement according to EBE characteristics and outcomes, with mean GPTS, WEMWBS, and UES scores estimated, stratified by engagement level.

## Results

### Discover

#### Wearable Technology

Desk research identified a range of wearable technologies that were easily accessible and commercially available. Considered solutions for the augmentation of SloMo included: wrist-worn bands and smartwatches, headsets, skin-adhered patches, in-earphones, clothing-embedded sensors, and smartphone software. The Spire Stone, a clothing-adhered biosensor commercially available until 2020, was selected to augment SloMo (Figure 1A) [36]. The Spire Stone had a publicly available API that enabled integration with the SloMo app, was financially feasible, and was considered discrete to wear relative to other devices.

**Figure 1.**
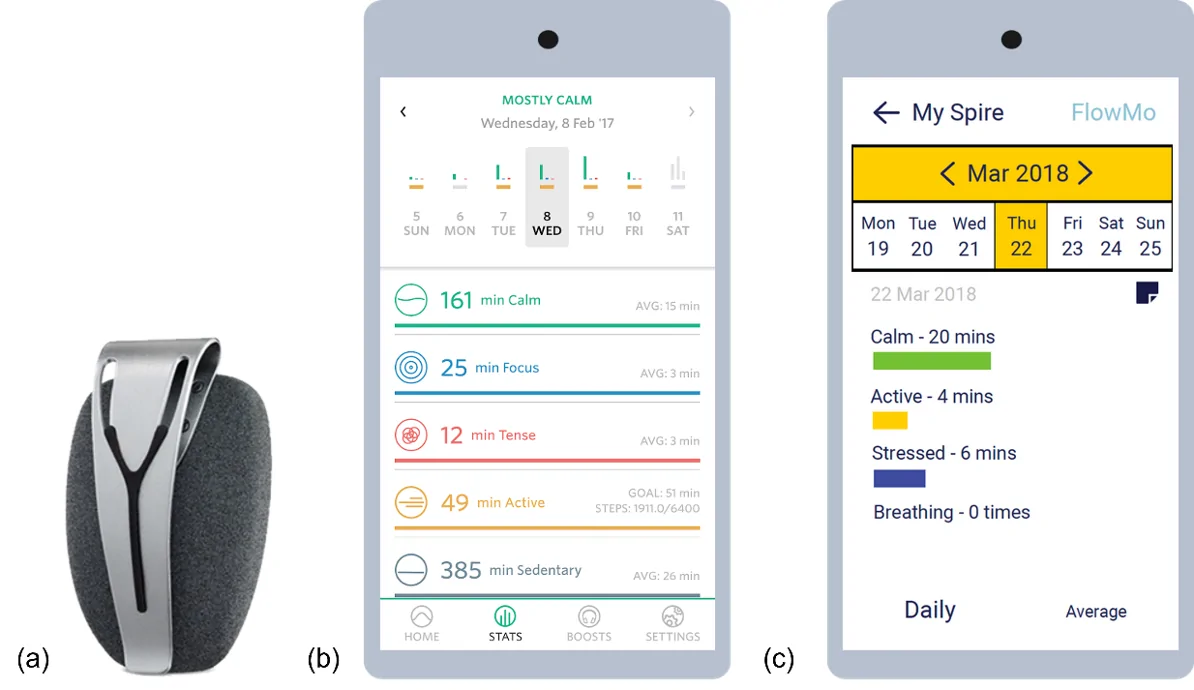
(A) Spire clothing-adhered biosensor; (B) example nonadapted Spire smartphone app interface; (C) example SloMo app-My Spire interface.

The Spire Stone continuously monitored respiratory rate variability and compared this to the individual’s median values after a period of calibration. Significant and sustained changes were interpreted as signals of cognitive-emotional state changes [37]. Feedback summarizing one-minute state intervals was presented to the wearer via a linked smartphone app, labeled as: “calm” (slow and consistent breathing), “focus” (moderately fast and consistent breathing), and “tense” (fast and erratic breathing) [18]. The device recorded the number of steps, inferring “active” compared to “sedentary” minutes. The wearer was also delivered in situ biofeedback notifications through device vibrations, to alert to sustained periods of states or sedentary behavior, aiming to facilitate self-regulation of stress levels. The Spire Stone was used previously to augment stress management interventions [18], and has demonstrated acceptability and usefulness [38]. Spire has reported strong correlations between their devices and “gold standard” medical device measurement of respiratory rate [39].

The nonadapted, commercially available Spire mobile app interface (Figure 1B) was also installed on the handsets provided to EBEs. Therefore, during the user testing, EBEs had the option to use either the Spire or SloMo wearable app, depending on their needs. Hereafter, we use “the technology” to refer to the integrated Spire Stone and linked SloMo wearable app.

#### Co-Design Feedback

Key themes regarding user needs related to acceptability, usability, and usefulness were identified from the EBEs’ initial impressions of the technology. Illustrative quotes are presented in Table 2.

**Table 2.** Illustrative quotes representing EBE feedback on acceptability, usability, and usefulness for phase 1 (discover).

Quote ID	EBE^a^	Feedback
Acceptability		
1	2	Understanding can be useful […] validating your experiences: this is why you’re feeling this way
2	3	I guess technology can make it more accurate to read how stressed you are and people who do not notice how they are feeling this could be a good way
3	8	Found the Spire app quite confusing to look at and said there were too many things going on, this put him off using it again
4	1	If someone was more stressed, it might annoy that person and highlight their anxieties for them
5	6	…Unhelpful to notice that she was feeling anxious as this would make her feel more anxious
6	1	Found the vibrations difficult to manage at work
7	3	Would use it more if [I] had more routine in [my] life, and at the moment [I’m] avoiding stressful situations and [do] not go out much so there is no point in wearing it when at home.
8	6	I trust you guys that you won’t give me something with lasers in
9	2	[The Spire] looks like a microphone […] it might be recording
10	3	…can be hacked, and people can use if for harmful reasons to induce stress from the technology itself.
Usability		
11	10	There are too many alerts […] but it’s not clear what the alert was for.
12	6	People who are worse at technology… or in the height of anxiety might not be able to know which are buttons or not, so could be made clearer.
Usefulness		
13	3	I don’t notice that I’m tense when I’m doing certain things, but it appears that at times I’m flustered, which is helpful to know
14	4	When you are paranoid, you are in a vacuum so a bubble [notification] coming up breaks that vacuum
15	5	…like the simplicity [of FlowMo], puts me in a calm state […] you take a pill for a headache and a breath for anxiety.
16	7	[FlowMo] stops me going over the edge and calm down.
17	9	Maybe I’m just so used to feeling tense that it doesn’t bother me that much
18	1	It wasn’t that helpful to be told multiple times by the spire that you’re ‘feeling tense’ and found it quite frustrating [it] was still ‘feeling tense’ after having done the breathing exercise

a EBE: expert by experience co-designer.

##### Acceptability

At baseline, most EBEs (n=7) perceived value in becoming more aware of emotions through monitoring of physical sensations (example quotes; Q 1‐2). Three EBEs enjoyed being able to track progress and receive feedback and valued visually simpler displays to reduce confusion (Q3). Some (n=3), however, noted that increasing focus on stress levels through the technology could unhelpfully exacerbate difficulties (Q4-5). Two EBEs cited difficulty integrating into current routines, conversely either due to busyness (Q6) or limited activity levels (Q7). While the majority did not report concerns about the trustworthiness of the technology (Q8), 2 shared suspicions that there could be malicious intentions behind it (Q9-10). Nonetheless, both remained willing to continue using the device within the study.

##### Usability

Though most felt sufficiently competent in using the technology and reported they found it easy to use, two raised concerns that those less confident may have difficulties. After initial use, some reported a lack of clarity from the app and difficulty knowing what Spire Stone vibrations were signaling (n=2, Q11). One EBE described difficulty initiating the FlowMo breathing exercise when experiencing distress (Q12). Five reported issues with the technology that impacted use: the need for a second smartphone handset, poor battery life, and issues with synchronization between the Spire Stone and the app.

##### Usefulness

EBEs (n=6) described how using the technology to identify when they were feeling stressed supported self-management (Q13-14), including use of the SloMo app’s wider features (n=6). Positive feedback was given on breathing exercises (n=6) in SloMo (FlowMo) or the Spire app, and GoMo (n=3), presenting an opportunity for relaxation, distraction, and prevention of worries worsening (Q15-16). Recommendations were given for the SloMo app to have further opportunities for relaxation and distraction, such as through calming sounds, music, or visuals, and games to have more variation (n=3). Monitoring and goal setting for activity levels within the Spire app were viewed as positive and motivating by some (n=3). While most (n=5) noted some accurate detection of difficult moments, 3 noted discrepancy and the technology overreporting stress, hypothesized to be due to desensitization by one EBE (Q17). In addition, the technology was noted to lack responsivity to efforts to reduce stress levels (n=2, Q18).

### Define and Develop

Insights regarding user needs from the Discover phase were reviewed, synthesized and prioritized into 7 problems to be addressed through the co-design work (Table 3). Iterative development sprints enabled the testing and refinement of solutions within the SloMo wearable app interface (Table 3, Figure 1C).

**Table 3.** Redefined problems identified through phase 2 (define) and associated design solutions tested in phase 3 (develop).

Problem	Design solution to meet user requirements
Self-monitoring and self-management of stress can be challenging and effortful	The SloMo-Spire integration should be easy to use and access, through clear controls, navigation, and language. A diary function for note-taking and goal-setting supports users in self-management, monitoring, and motivation.
A focus on the experience of high stress can increase anxiety for users	Increased user controls enable the option to turn off “stress” state display and feedback. Notifications encourage users to recognize changes in emotion, rather than focusing solely on negative states.
Frequent notifications to alert to changes in emotional state through vibration lack clarity and meaning	Users can adjust notification time and frequency settings; users are provided with clear visual feedback on respiratory data. Vibration alerts have a corresponding visual, written app notification to prompt user action (ie, slowing down thoughts, FlowMo, or GoMo).
Feedback on emotional states that is misaligned with the user’s own perception can be experienced as frustrating, confusing, and detrimental to trust	Simplification of data feedback to combine “focus” and “tense” states into one “stressed” state. SloMo app notifications neutrally prompt user reflections on current safety and distress to facilitate awareness and self-management.
Coping enhancement activities without variation and personalization offer limited opportunity for relaxation and/or distraction	Expansion of coping enhancement activities through the addition of sounds and music to FlowMo, and additional difficulty settings in GoMo.
Digital mental health intervention software may present challenges for those who are less technologically confident or experiencing acute anxiety	Users should be offered a clear demonstration, and additional support or repetition may be required to ensure confidence in use. The SloMo wearable app interface requires clear, easy-to-use buttons to activate coping activities.
Wearable technology can be a source of suspiciousness and paranoia	Users should be offered information regarding the purpose, function, and security of the technology, tailored according to individual concerns. This information should be easily accessible within the SloMo software.

### Deliver

#### Qualitative Feedback

Illustrative quotes are presented in Table 4.

**Table 4.** Illustrative quotes representing EBE^a^ feedback on acceptability, usability, and usefulness for phase 4 (deliver).

Quote ID	EBE	Feedback
Acceptability		
19	6	Was very excited to see her suggestions about relaxing noises being added […] really liked the forest noises in particular, as it reminded her of home
20	4	Finding it more enjoyable using the updated version […]using both levels on GoMo
21	5	Stopped using and made him worry that information might be broadcast to others […] He liked the updated app. The ‘About SloMo’ section reassured him [about] confidentiality and security, he would read for reassurance.
22	9	Has become habit to put on in the morning and play about with later on to see how he has been feeling
23	2	More positive about explaining the device and what it does to other people, and them being able to accept it
24	7	‘Cause if the person’s like me it would help them 100%. ‘Cause like, I feel alright since I’ve been using it. It’s helped me through all the bad times I’ve been using it, it’s helped me.
Usefulness		
25	6	The app/device itself is not enough to remove the distress but it is great for helping notice her distress and take extra steps to alleviate it. […]
26	4	Spire will help clients deal with things better in between seeing their CPN or make them see CPN less often by supporting people in self-managing experiences
27	6	When you’re on your own, it makes you feel like […] someone is actually there to say ‘you can do these things.’
28	10	Learning [the] breathing without looking at the app […] can see it in his mind’s eye and after a while won’t need the app.
29	2	Found it the most frustrating that the app couldn’t pick up on the distress his voices were expressing. He felt like the app made him look like a liar, as the distress experienced by the voices wasn’t being picked up.

a EBE: Expert by Experience co-designer.

##### Acceptability

EBEs commented on liking the updated SloMo wearable app interface with regards to its simplicity and visual appeal (n=3). Changes made through the co-design process received positive feedback, specifically regarding the addition of relaxing sounds to FlowMo (n=2, Q19) and difficulty levels to GoMo (n=4, Q20) to better facilitate coping in response to wearable prompts. Some EBEs (n=4) expressed discomfort or inconvenience related to the wearable device type, with preference for alternatives (eg, wrist-worn and smartphone embedded). While 4 noted concerns related to intrusiveness, data privacy or app security, availability of in-app related information to provide clarity and reassurance was valued (Q21). EBEs described developments over time such as successful integration into daily life and habitual use (n=4, Q22), and becoming increasingly comfortable discussing the technology with loved ones (n=2, Q23). Six EBEs perceived that others with similar difficulties would be likely to benefit (Q24). The UES demonstrated overall positive ratings of acceptability (mean 72.7%, SD 28.1%) and enjoyment (mean 68.3%, SD 20.6%).

##### Usability

Several EBEs (n=4) commented on the ease of use of the technology. However, one user continued to feel they lacked technological skills or interest to benefit and wanted the app to be more interactive or include 2-way communication with a person (eg, therapist) for support (P2). Limitations of the technology continued to impact experience, such as synchronization issues (n=2), inconvenience of requiring two handsets (n=1), and battery or charging issues (n=1). Responses to the UES demonstrated overall positive usability (mean 68.3%, SD 17.4%).

##### Usefulness

The majority of EBEs viewed the technology to be effective in facilitating self-management of difficulties (n=8) and reducing distress (n=4). While several noted the worn device did not solve the difficulty itself (n=3), it instead enabled them to become aware, prompting them to “do something differently,” such as using the SloMo app’s features (n=4), or engaging in other strategies (Q25). Four EBEs likened the app to an interim alternative to support from others (eg, a therapist, or community psychiatric nurse, Q26-27). Some described how they had begun to generalize learning to other contexts when not using the app (n=3, Q28). Three EBEs found there continued to be inaccuracies between the wearable feedback and their own perception, and for one this related specifically to the experience of hearing voices (Q29). Across the whole group, changes in outcomes were highly variable for both paranoia (total GPTS change mean −3.0, SD 18.1) and well-being (WEMWBS change mean −1.6, SD7.44).

##### Engagement

EBE feedback indicated varying levels of engagement with the technology through the co-design period. Three were categorized as “higher” usage, indicating they wore the Spire Stone and engaged with the technology for most days throughout the co-design period (“wearing the spire device every day from the moment she wakes up to moment she goes to bed,” P4). Four indicated use for several days each week, or most weeks of the period and were considered “mid” usage. Three had limited engagement, using the technology for only a small proportion of the 8-week period (“didn’t really use the app at all in last few weeks,” P2).

EBE characteristics in relation to usage were explored to facilitate understanding of who may engage with and benefit from wearable augmentation of digital interventions, such as SloMo (Table S3 in Multimedia Appendix 1). Both female EBEs reported higher usage, however, each engagement group otherwise represented a range of ages, ethnicities, and smartphone confidence. As shown in Table 5, higher frequency users reported the most severe paranoia at baseline, while well-being scores were slightly lower for low frequency users at baseline.

**Table 5.** Mean pre- and postintervention scores for usefulness and acceptability outcome measures, by level of self-reported engagement.

Outcome, mean (SD)	Engagement level					
	Lower (n=3)		Mid (n=4)		Higher (n=3)	
	Baseline	8-week	Baseline	8-week	Baseline	8-week
Paranoia (GPTS^a^ Total)	77.3 (25.4)	82.3 (22.8)	69.0 (27.6)	74.5 (5.5)	84.3 (35.9)	62.0 (21.2)
Paranoia: referential ideas (GPTS Part A)	38.0 (10.4)	41.3 (10.4)	33.0 (13.5)	33.25 (12.8)	44.0 (18.1)	30.7 (11.0)
Paranoia: persecutory ideas (GPTS Part B)	39.3 (15.0)	41.0 (12.5)	36.0 (14.1)	41.25 (12.9)	40.3 (18.8)	31.3 (10.2)
Well-being (WEMWBS)^b^	34.7 (3.2)	36.3 (2.5)	41.25 (4.5)	38.8 (5.2)	42.7 (7.4)	39.0 (21.2)
Satisfaction% (UES^c^ Total)	—^d^	47.3 (22.0)	—	75.5 (4.4)	—	84.0 (13.0)
Enjoyment% (UES)	—	45.6 (18.4)	—	77.5 (5.7)	—	78.9 (20.4)
Usability% (UES)	—	47.5 (10.9)	—	73.8 (7.2)	—	81.7 (13.8)
Acceptability% (UES)	—	48.9 (42.3)	—	75.8 (13.4)	—	92.2 (6.9)

a GPTS: Green et al, [29] Paranoid Thought Scale (Subscale scores range from 16‐80, total scores 32‐160, higher scores indicate more severe symptoms).

b WEMWBS: Warwick-Edinburgh Mental Well-being Scale (scores range from 14‐70, higher scores indicate better well-being).

c UES: User Experience Survey (scores reported as percentage agreement for each scale).

d Not applicable.

Level of engagement differed according to the technology’s usefulness and acceptability to the user. Those who used the technology most also experienced improvement in total paranoia scores at 8-week follow-up (clinically observable response, n=2; reliable change; RC; n=1; see Table S3 in Multimedia Appendix 1). In contrast, a pattern was observed that lower and mid frequency users reported a slight deterioration, although this did not meet threshold for reliable change. Well-being scores remained consistent overall regardless of engagement, however one higher, and one mid frequency user reported a reliable deterioration in well-being. Similarly, acceptability data provided by UES responses aligned with self-reported usage, such that those that engaged more were also most satisfied, while low frequency users were least.

## Discussion

### Principal Findings

This study is the first to explore the user experience of wearable technology integrated with an existing digital therapy for paranoia in people with psychosis. A minimal viable product was first developed, comprising a user interface that integrated the wearable technology with the SloMo mobile app. Most users found the developed technology to be usable, acceptable, and enjoyable to use (mean agreement>68%, SD 20%). Some EBEs reported using the technology frequently and had higher overall user satisfaction (agreement 84%, SD 13%), finding the wearable to be discrete, secure, robust, accurate, and useful, and reported reductions in paranoia. For other EBEs who described using the technology less often, modest user satisfaction was reported (47%, SD 22% to 76%, SD 4%) and there was no marked impact on paranoia. There was encouraging early evidence that, for some, wearables might promote engagement with a therapy app to support management of paranoia, at least in the short term, and even in the absence of therapist support.

The findings highlight the potential for responsive, wearable technology to improve engagement. Almost all EBEs (8/10, 80%) reflected that when using the Spire device, they also used the SloMo app more frequently. There are known challenges for engagement and retention in digital mental health interventions over time [40,41]. Strategies that are tailored and prompt intervention engagement when it is most needed may optimize effects and enhance subjective usefulness. The longer-term effectiveness of SloMo is not yet known and is currently being evaluated in an implementation-effectiveness study (ClinicalTrials.gov ID NCT06568081). The current study suggests that wearable integration may be a plausible way of encouraging ongoing use if engagement is found to decline after formal therapy completion.

For people with psychosis, to date, wearable technology has most commonly been used as a tool for assessment, monitoring, and diagnosis [42]. There is great interest in the potential of digital phenotyping (ie, passive sensing) through smartphones to detect and prevent relapse [43]. However, our inclusive, user-centered design research suggests that people want technology to provide secure, accessible, and responsive support to assist self-management, and are reluctant for this to involve the sharing of personal data without sufficient clarity and reassurance. This is consistent with the findings of a study exploring perspectives of people with psychosis on passive sensing, highlighting the importance of fully informed consent, choice, and autonomy [44]. Similarly, despite an initial focus on monitoring stress in this study, EBE feedback demonstrated this emphasis to be undesirable to some. Instead, a preference was shared for the wearable to help increase awareness of “calm” and “active” states, and the associated activities and strategies that support this. Wearable technology including active biofeedback, not solely passive monitoring, may be key to realizing the promise of digital health in innovating the treatment of psychosis (see Figure 2).

**Figure 2.**
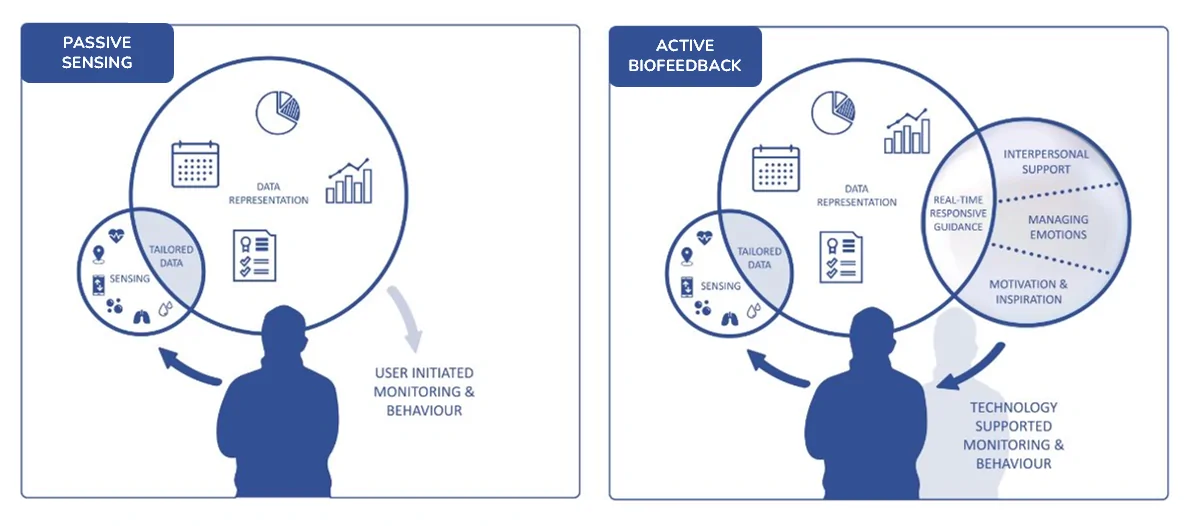
Wearable therapeutics offering biofeedback as a preferred method by EBE co-designers for supporting monitoring of symptoms and behavior change, relative to passive sensing only.

The participating EBEs represent a diverse group in relation to age, ethnicity, technological confidence, and paranoia severity. Engagement and user experience were not impacted by these factors. Notably, as a group, people who reported using the technology most had the most severe paranoia at the outset. While some EBEs expressed concerns about the trustworthiness and safety of wearable technology, it did not deter them from continuing to use it. Reassurance, gaining familiarity over time, and availability of accessible written information regarding data privacy and security were viewed as helpful solutions to these concerns. Concerns regarding “service users feeling suspicious or paranoid” are cited as the most common barrier to mental health professionals recommending the use of digital mental health interventions (38). This study demonstrates that this concern may risk preventing access for those who could benefit.

In keeping with the early stage of the work, technological challenges were common, with the group reporting technical issues (7/10, 70%), usability problems (5/10, 50%), discomfort (4/10, 40%), and discrepancy with their own perceived emotional state (4/10, 40%). Advancements in wearable technologies may alleviate some of these challenges. However, an exacerbated discrepancy between self-reported and physiological stress levels in people with psychosis has been reported previously [45]. Biofeedback should be suggested cautiously, for example, “You may be feeling stressed; would you like to slow down?” This approach avoids damaging the person’s trust in the technology.

### Limitations and Future Directions

The EBE co-designers involved in this study represent a small, but diverse group of individuals with regards to age, ethnicity, technological confidence, and attitudes towards technology. All EBEs had engaged in SloMo therapy prior to involvement and had completed therapy with high rates of adherence. To this extent, the study’s findings represent the views of people who engaged well, with prior knowledge of SloMo, and may differ from those who may be less engaged with digitally supported talking therapy. Furthermore, as the same group of EBEs were involved throughout the co-design process, acceptability ratings may be higher due to the implementation of their own recommendations for improvements. Conversely, EBEs were not involved in the initial selection of the Spire Stone wearable device. Consultation regarding user priorities for the device at an earlier stage may identify an alternative, more acceptable device to users and may facilitate improved engagement.

The experiences and outcomes reported were highly variable. Females were underrepresented in the group; however, both female EBEs used the technology frequently, reported high satisfaction, and benefitted in terms of paranoia improvements. Higher engagement and satisfaction with digital interventions for psychosis by female users has been noted previously [13], and was similarly found in the SlowMo RCT [1]. This indicates a clear need for development work to further optimize the wearable augmentation of SloMo to ensure acceptability for a wider range of people, particularly nonfemale users.

Baseline data was collected subsequent to the 24-week follow-up for the SlowMo RCT. In the RCT, participants typically experienced benefits to paranoia and well-being following SloMo therapy that were maintained at follow-up [1]. To this extent, most EBEs in the current study commenced participation having already achieved clinical benefit from SloMo therapy. Less engaged users experienced fluctuations in paranoia, as is expected in this population [46], though levels remained in keeping with the SlowMo treatment group average at 24-week follow-up. Furthermore, the fluctuation for these groups did not exceed the recommended minimally clinically significant response used in meta-analyses of CBTp [31]. Regardless, potential iatrogenic harms should continue to be explored in future work.

Exploratory post hoc investigations in relation to patterns of engagement were conducted based on coding of qualitative descriptions by the EBEs. The patterns observed correspond to the objective data collected in this study, such that use of a digital health technology is influenced by a complex system of interacting components including acceptability (eg, beliefs, knowledge, and affective attitude), usability, and usefulness [20]. Objective measurement of engagement (eg, technical usage data) and a priori operationalization of engagement levels would strengthen future similar investigations.

Reflective of the technology available at the time of study delivery, the reliability for “stress detection” was limited: the device could identify changes in breathing patterns but was insufficient in accurately determining whether these changes reflected genuine distress. Accordingly, the Spire Stone was reported as inaccurate in stress signal detection by several EBEs, impacting the perceived usefulness of the integrated technology. Advancements in wearable technology are rapidly developing [47,48] and may offer an improved solution that enhances trust, usefulness, and therefore the overall acceptability of mental health interventions.

### Conclusions

This study demonstrates that wearable technology can be integrated into SloMo, a digitally supported therapy for psychosis. Involving those with lived experience in the development of digital mental health interventions is crucial to ensuring alignment with user needs. While standalone, passive monitoring of stress was not viewed as useful by EBEs, people were positive about the potential utility of integrating wearables with digital interventions to facilitate self-management of difficulties. People may use therapy apps more frequently when wearable devices are added, which is important because the experience and outcomes of current psychological therapies for psychosis need to be improved. However, wearable technology outcomes currently remain variable. Development work is needed to broaden acceptability, usability, and usefulness for a wide range of people.

## Supplementary material

10.2196/91188 Multimedia Appendix 1 Semi-structured interview topic guides; User Experience Survey; engagement coding description; Reliable Change Indicator calculation; individual EBE characteristics.

## Abbreviations

CBT: cognitive behavioral therapy
CBTp: cognitive behavioral therapy for psychosis
EBE: expert by experience
GPTS: Green Paranoid Thought Scale
HCD: human-centered design
iHCD: inclusive human-centered design
RCI: reliable change index
RCT: randomized controlled trial
UES: User Experience Survey
WEMWBS: Warwick-Edinburgh Mental Wellbeing Scale
