# Supplementary material for: Integrating Wearable Technology in Digital Therapy for People With Psychosis (SloMo): Mixed Methods Co-Design and Feasibility Study
Source: JMIR Hum Factors. 2026 Oct 2;13:e91188. doi: 10.2196/91188 (PMC13633062; doi:10.2196/91188)
Supplement: Supplementary file 1 [file humanfactors-v13-e91188-s001.docx]

**Integrating wearable technology in digital therapy for people with psychosis (SloMo): Mixed methods co-design and feasibility study**

**Supplementary Materials**

Contents

[Section 1: Semi-structured Interview: Attitudes Towards Monitoring Stress and Technology Use:	2](#_Toc238725915)

[Section 2: Semi-structured interview schedule: User feedback	3](#_Toc238725916)

[Section 3: User Experience Survey	4](#_Toc238725917)

[Section 4: Engagement coding	5](#_Toc238725918)

[Section 5: Reliable Change Indicator calculation	6](#_Toc238725919)

[Section 6: Individual EBE characteristics and outcomes	7](#_Toc238725920)

## Section 1: Semi-structured Interview: Attitudes Towards Monitoring Stress and Technology Use:

Do you notice how stressed you are feeling?

- If yes, what do you do to notice how you are feeling?
  - If yes, what makes it easier or harder to notice how you are feeling?
  - If yes, in what ways is it helpful or unhelpful to notice how you are feeling?
- If no, how come you do not notice how you are feeling?
  - If no, what would make it easier or harder to notice how you are feeling?
  - If no, in what ways could it be helpful or unhelpful to notice how you are feeling?

1. What do you think about using technology to monitor your physical or mental health?
2. Have you ever used technology to monitor your physical or mental health?

- If so, what have you done?  How did you find it?

3. What do you think about using technology to monitor stress?
4. In what ways could using technology to monitor stress be helpful or unhelpful?
5. Do you have any concerns about using technology to monitor stress?

- If yes, what would help you feel better about using technology to monitor stress?

## Section 2: Semi-structured interview schedule: User feedback

1. What do you think about it?

2. In what ways is it helpful?

3. In what ways is it less helpful?

4. Are there any difficulties or barriers to using it?

5. Is it relevant to your difficulties?

6. Would you find it helpful for managing upsetting experiences?

7. What impact might it have, if any?

8. Is there anything missing?

9. Is there anything you would change?

10. Is there anything you particularly enjoyed?

11. Do you have any other concerns?

12. Would you use it in the future?

13. What do you think other people would think about it?  In what ways might their views be similar or different to yours?

## Section 3: User Experience Survey

We are interested in your experience of using the [SloMo wearable] mobile app.  Your feedback is very important for helping us to improve the app, so please be as honest as possible.

Please rate each of the items below on a scale of 0 = ‘totally disagree’ to 10 = ‘totally agree’.

Rating (0 to 10)

1. I enjoyed using Spire
2. Spire was easy to use
3. I think Spire would help me to manage my difficulties better
4. I would recommend Spire to other people with similar difficulties to me
5. Spire was too complicated to be helpful to me
6. Spire felt relevant to me and my problems
7. The information on Spire was clear and easy to understand
8. I just couldn’t get to grips with Spire
9. Spire was fun and engaging
10. I felt frustrated using Spire

1, 9, 10* – enjoyment

2, 5*, 7, 8* – usability

3, 4, 6 - acceptability

* item reverse scored

## Section 4: Engagement coding

| ***Supplementary Materials Table 1.*** *Engagement coding description with participant examples.* | | | |
| --- | --- | --- | --- |
| **Engagement level** | **Descriptor** | **Participant** | **Example** |
| Lower | Evidence of infrequent or intermittent use of the technology, equivalent to a minority (<33% or up to 18 days) of the testing period. | P1 | ‘used the spire for around 10 days’ |
|  |  | P2 | ‘didn’t really use the app at all in last few weeks’ |
|  |  | P8 | ‘did not use beyond 1 week assessment’ |
| Mid | Evidence indicating fluctuating or partial use of the technology, equivalent to half (>34% & <66% or 19 to 37 days) of the testing period. | P3 | ‘he did not use the SlowMo Wearable app much as he tended to use the Spire because he could have it on his phone’ |
|  |  | P5 | ‘Said had been using Spire every day, to help when tense, so can do something about it. But actually, hadn’t been using spire that much.’ |
|  |  | P9 | ‘Mixed usage of the spire device and apps since [week 1], stating that he did not wear/use for a couple of weeks when feeling “unwell”’ |
|  |  | P10 | ‘wore it 3 to 4 times in the last week’ |
| Higher | Frequent use sustained throughout, equivalent to the majority (>67% or 37 to 56 days) of the testing period | P4 | ‘wearing the spire device every day from the moment she wakes up to moment she goes to bed’ |
|  |  | P6 | ‘Enjoyed using spire, wore it most of the time’ |
|  |  | P7 | ‘Every day – charge it in the morning and go on it in the day.’ |

## Section 5: Reliable Change Indicator calculation

| Supplementary Materials Table 2. Test-retest reliability, sample means and standard deviation used for reliable change indicator calculation | | | |
| --- | --- | --- | --- |
| Measure | Test-retest reliability: Cronbach’s alpha | Mean (Standard deviation) | Reliable Change Index Value |
| GPTS Total | 0.90^a^ | 101.9 (29.8) ^a^ | 26.1 |
| WEMWBS | 0.93^b^ | 44.6 (12.8)^b^ | 9.39 |

Note. GPTS = Green Paranoid Thought Scale; WEMWBS = Warwick Edinburgh Mental Wellbeing Scale. Test-retest reliability and sample data were obtained from: ^a^Green *et* al., 2008 ; ^b^Freeman *et al*., 2019.

## Section 6: Individual EBE characteristics and outcomes

| Supplementary Materials Table 3. Individual EBE demographic and baseline characteristics, and pre-post change from baseline to 8-week follow-up. | | | | | | | | | | |
| --- | --- | --- | --- | --- | --- | --- | --- | --- | --- | --- |
| **EBE** | **Usage** | **Gender** | **Age group** | **Ethnic background** | **SlowMo1 smartphone confidence (%)** | **SlowMo1 GPTS-total change** | **SlowMo1 WEMWBS change** | **SloMo-Spire GPTS-Total change** | **SloMo-Spire WEMWBS change** | **UES satisfaction (%)** |
| 1 | Lower | Male | 35 - 49 | Hispanic or Latin American | 73 | -39 | 11 | 14.00 | 3.00 | 58.0 |
| 2 | Lower | Male | 35 - 49 | Black^a^ | 11 | 1 | -2 | 14.00 | .00 | 62.0 |
| 3 | Mid | Male | Below 35 | White | 95 | -40 | 2 | 8.00 | -4.00 | 70.0 |
| 4 | Higher | Female | 50 + | White | 90 | -16 | 3 | -39.00^c,d^ | 9.00 | 97.0 |
| 5 | Mid | Male | 35 - 49 | White | 60 | -1 | 3 | 17.00 | .00 | 74.0 |
| 6 | Higher | Female | 35 - 49 | Asian^b^ | 40 | -45 | -1 | -11.00 | -3.00 | 84.0 |
| 7 | Higher | Male | 50 + | Black | 50 | -43 | 13 | -17.00^d^ | -17.00^c^ | 71.0 |
| 8 | Lower | Male | Below 35 | Black | 80 | -55 | -5 | -13.00 | 2.00 | 22.0 |
| 9 | Mid | Male | 35 - 49 | White | 81 | -9 | 1 | -11.00 | -10.00^c^ | 79.0 |
| 10 | Mid | Male | 50 + | Black | 90 | -15 | 8 | 8.00 | 4.00 | 79.0 |
| Notes. ^a^‘Black’ includes Black, Black British, Black Welsh, Caribbean or African; ^b^‘Asian’ includes Asian, Asian British, Asian Welsh; ^c^Reliable change criterion met; ^d^Clinically observable response criterion met  GPTS = Green et al., Paranoid Thoughts Scale (Scores range from 32-160, higher scores indicate more severe symptoms). WEMWBS = Warwick-Edinburgh Mental Well-being Scale (scores range from 14-70, higher scores indicate better wellbeing). UES = User Experience Survey (scores reported as percentage agreement). | | | | | | | | | | |
